# Amplification of PIP3 signalling by macropinocytic cups

**DOI:** 10.1042/BCJ20170785

**Published:** 2018-02-14

**Authors:** Robert R. Kay, Thomas D. Williams, Peggy Paschke

**Affiliations:** MRC Laboratory of Molecular Biology, Francis Crick Ave., Cambridge CB1 0QH, U.K.

**Keywords:** *Dictyostelium discoideum*, macropinocytosis, PIP3, protein kinase B, Ras

## Abstract

In a role distinct from and perhaps more ancient than that in signal transduction, PIP3 and Ras help to spatially organize the actin cytoskeleton into macropinocytic cups. These large endocytic structures are extended by actin polymerization from the cell surface and have at their core an intense patch of active Ras and PIP3, around which actin polymerizes, creating cup-shaped projections. We hypothesize that active Ras and PIP3 self-amplify within macropinocytic cups, in a way that depends on the structural integrity of the cup. Signalling that triggers macropinocytosis may therefore be amplified downstream in a way that depends on macropinocytosis. This argument provides a context for recent findings that signalling to Akt (an effector of PIP3) is sensitive to cytoskeletal and macropinocytic inhibitors.

One of the most rapid and dramatic effects of growth factors on responsive cells is to stimulate their actin cytoskeleton into a brief period of ruffling and macropinocytosis [1]. This can be mimicked with oncogenic Ras [2] and blocked by phosphoinositide 3-kinase (PI3K) inhibitors [3], yet its physiological purpose is not clear. Why should this signalling pathway trigger the actin cytoskeleton? At the same time, including in a recent issue of this journal, there are reports that signalling to PI3K can be amplified in a way that depends on the actin cytoskeleton [4,5]. These two strands of work may be tied together by evidence that Ras/PIP3 signalling triggers not only macropinosome formation, but is also a spatial organizer of macropinocytic cups, which role we propose necessitates a degree of signal amplification within the cup.

Macropinocytosis is the large-scale imbibing of extracellular fluid into intracellular vesicles [6–9] (Figure 1). Macropinosomes form from cups several microns in diameter that are extended from the cell surface by a ring of protrusive actin polymerization beneath the plasma membrane. These cups eventually close, entrapping a droplet of medium in a vesicle. This vesicle is then trafficked within the cell so that lysosomal enzymes are added, the contents digested and useful molecules extracted. Macropinocytosis was the first form of endocytosis to be described, more than 80 years ago in early tissue cultures, where cells could be seen to entrap droplets of medium and transport them inwards. Macrophages are strongly macropinocytic, as are certain tumour cells, and it was envisioned even in the early days that, by taking up protein-rich serum, cancer cells might be feeding. Apart from this role in cancer cell nutrition [10], macropinocytosis is used by immune cells to take up antigens, provides an entry route for pathogens and drugs, and a means for neurodegenerative disease to spread from cell to cell [11]. Yet, despite its evident importance, macropinocytosis is one of the least understood of cell-biological processes.

**Figure 1.**
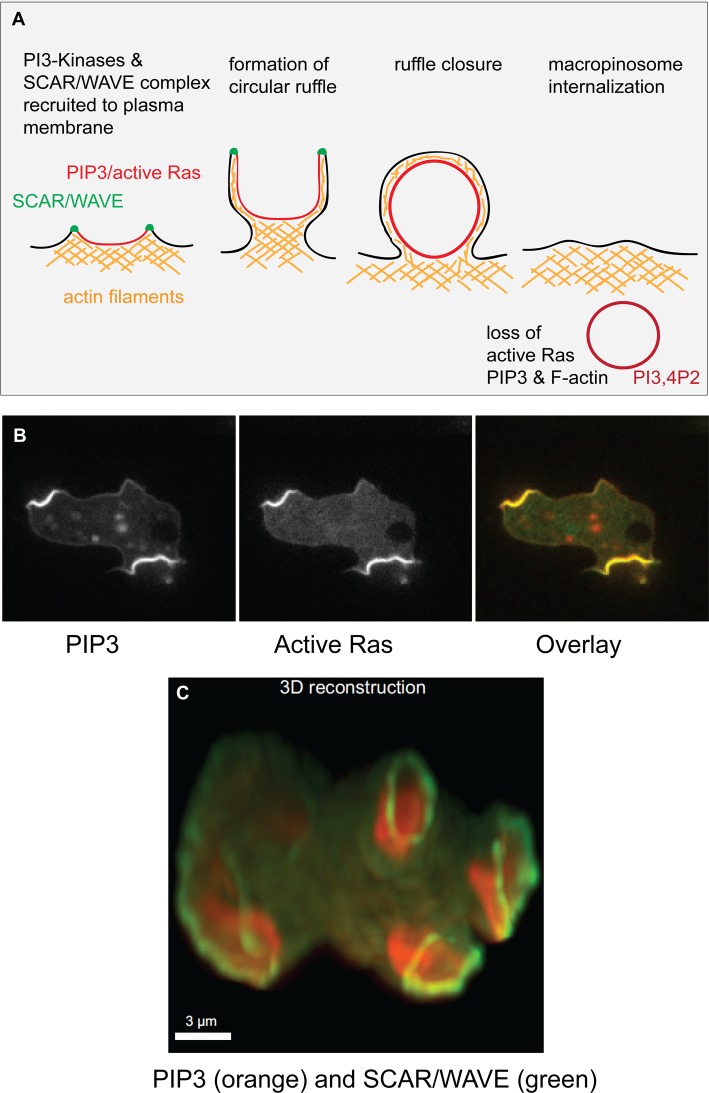
PIP3 and active Ras patches organize macropinocytic cups in the plasma membrane of *Dictyostelium* cells. (**A**) Schematic diagram of macropinosome formation, showing the extension of a circular ruffle organized around a patch of intense PIP3 and active Ras in the plasma membrane. The circular ruffle can be several microns in diameter and eventually closes to create a macropinosome, which loses its PIP3, active Ras and F-actin. The PIP3 is converted into PI3,4P2 and the vesicle is trafficked into the cell. This diagram is based on *Dictyostelium* work; in mammalian cells, the importance of PIP3 for macropinosomes is well established, but that of Ras and SCAR/WAVE less so. It should be noted that *Dictyostelium* phosphoinositides are chemically unusual, being ether-linked, plasmanylinositols [28] but appear functionally equivalent to their mammalian counterparts. (**B**) PIP3 and active Ras form coincident, intense patches in the plasma membrane. (**C**) The SCAR/WAVE complex (green) is recruited to the periphery of these patches (reported by PIP3, red) where it activates the Arp2/3 complex to trigger actin polymerization. This provides the template for the walls of a macropinocytic cup. The images show growing Ax2 cells either in section or 3D-rendered, expressing reporters derived from PH-CRAC for PIP3, RBD of Raf1 for active Ras and HSPC300 for the SCAR/WAVE complex. Taken from ref. [21].

Macropinocytosis probably evolved for feeding in single-celled organisms. It is used for this purpose today by certain amoebae, including the model *Dictyostelium discoideum*. Laboratory-adapted, axenic strains of *Dictyostelium* maintain a high rate of constitutive macropinocytosis that allows them to grow in liquid media [12–14]. This is independent of external signals and can be maintained by isolated cells in buffer (Thomas D. Williams, unpublished). The underlying cause of the increased macropinocytosis in axenic strains is deletion of the amoebal homologue of the RasGAP NF1 (neurofibromin) [15]. Loss of NF1 increases fluid uptake 10- to 20-fold, thus firmly implicating Ras as a positive effector of macropinocytosis. Similarly, type-1 PI3K are essential for efficient macropinocytosis, as shown by inhibitor experiments with mammalian cells and gene knockouts in *Dictyostelium* [16–18].

From a cell-biological perspective, one of the major questions concerning macropinocytosis is: how can cells form a ring of protrusive F-actin to make the walls of a macropinocytic cup? In the abstract, how can actin be persuaded to polymerize as a hollow ring several microns in diameter?

One clue comes from the observation in both mammalian and *Dictyostelium* cells that macropinocytic cups (or circular ruffles) form around patches of intense PIP3 accumulation, which can be visualized with fluorescent reporters, such as the PH-domain of Akt linked to GFP (Figure 1B). These patches coincide with patches of active Ras and Rac, forming a domain a few microns across of intense signalling within the plasma membrane [19–21]. PIP3 patches last throughout the lifetime of a circular ruffle, but PIP3 is lost from the membrane once the cup has closed and formed an intracellular vesicle [22]. There is no consensus yet as to how these PIP3 patches form, and indeed, this may differ in different cells but whatever the exact route, F-actin ruffles are pinned to their edges.

In macrophages, the cell surface has many linear ruffles, but a PIP3 patch only forms when one of these circularizes to form a circular ruffle [19]. How circular ruffle formation could trigger increased PIP3 production within it is not known, but it may be significant that the ruffle forms a diffusion barrier [23], which could trap and concentrate critical reactants. Scaffolding and cross-linking proteins within the circular ruffle may play a similar role. The very prominent PIP3 patches of the *Dictyostelium* plasma membrane can form *de novo*, expanding up from a small origin, or by splitting from existing patches. Strikingly, the SCAR/WAVE complex — which activates actin polymerization through the Arp2/3 complex — is recruited to the very edge of these patches (Figure 1C) [21]. The resulting ring of SCAR/WAVE potentially explains the formation of a ring of protrusive actin.

Although PIP3 patches form spontaneously in *Dictyostelium*, they are also triggered by extracellular cyclic-AMP (a chemoattractant in this organism) acting through a G-protein coupled receptor (GPCR), but with an unusual dose-responsiveness. As dose is increased the number of patches increases, but their size and intensity does not change. This suggests that once triggered, PIP3 patches are self-organizing [24]. Self-organization can imply a driving positive feedback loop, coupled with a negative feedback to prevent the whole system from becoming activated. Similarly, in mammalian cells, even with diffusion barriers around macropinosomes [23], it seems likely that strong positive feedback is required to sustain high local concentrations of diffusible molecules, such as PIP3. The postulated feedback loop might involve PIP3 itself (PIP3 stimulates PIP3 production) or a loop linking other points in the signalling pathway, perhaps involving Ras activation.

Thus, the proposal is that macropinocytic cups are shaped around intense patches of PIP3 and small G-protein signalling in the plasma membrane. These patches recruit the SCAR/WAVE complex to their edges, locally activating the Arp2/complex and stimulating a ring of protrusive actin polymerization. We further propose that the patches themselves are sustained by unusual dynamics involving positive feedback in Ras activation/PIP3 production and that these dynamics depend, in some way, on the integrity of the F-actin cup (Figure 2).

**Figure 2.**
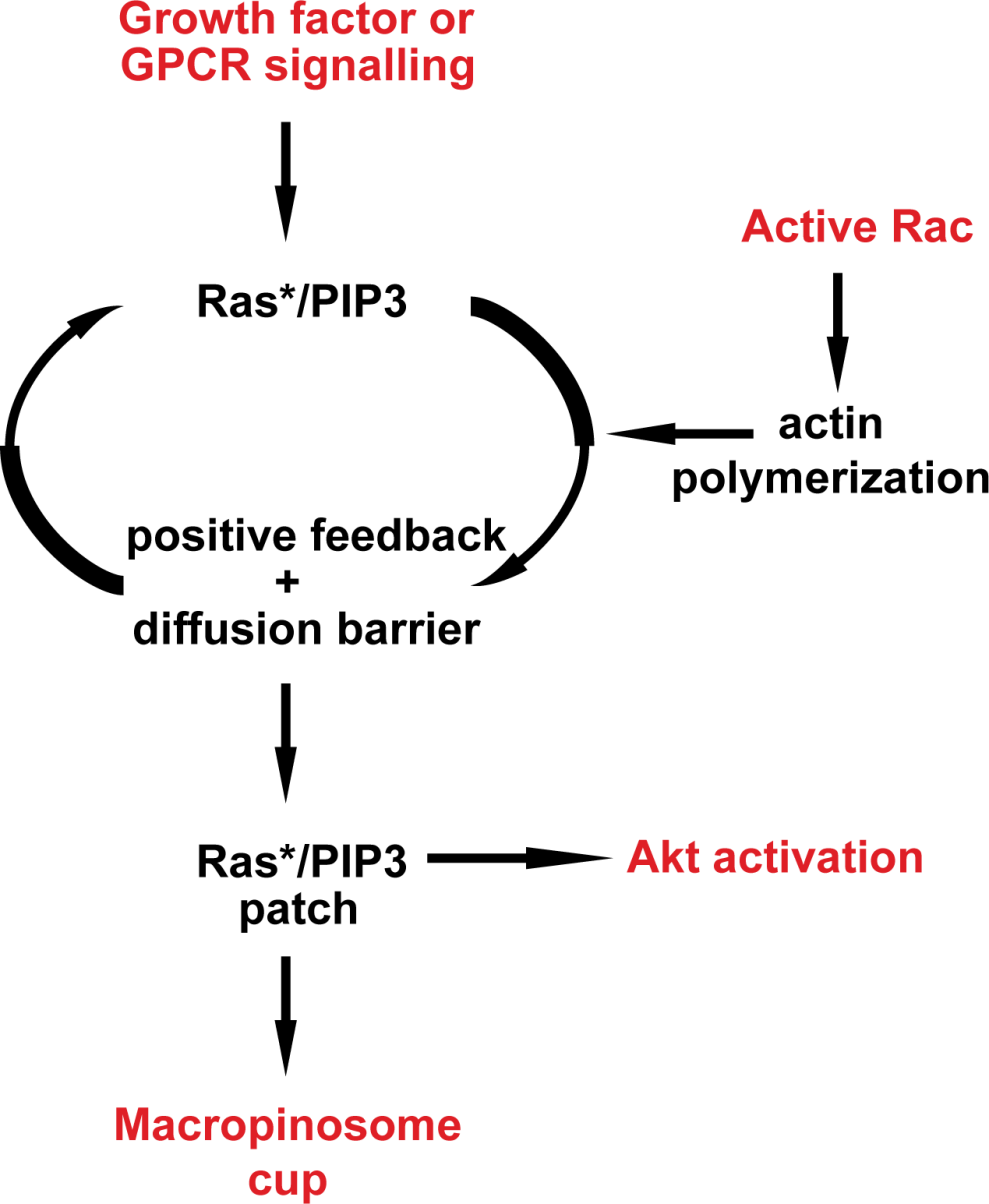
The proposed macropinocytic amplifier. The basic mechanism of the active Ras/PIP3 amplifier is unknown, but is assumed to depend on a positive feedback loop involving PIP3 and to require some function provided by the actin cytoskeleton. This requirement makes it sensitive to inhibitors of the cytoskeleton and of macropinocytosis and provides an input for active Rac through its well-established role in stimulating actin polymerization. An inhibitory process to restrain the positive feedback is also required, but not shown.

Macropinocytosis can be stimulated in mammalian cells by ligands acting through either tyrosine receptor kinases (RTKs) or GPCRs. Pacitto et al. [4] found that both macropinocytosis and the protein kinase, Akt, are stimulated in macrophages by the cytokine CXCL12 (a GPCR ligand). As expected, inhibiting PI3K blocks Akt activation, which depends on PIP3; but surprisingly so does inhibiting macropinocytosis. The macropinocytosis inhibitors used in this work act in completely different ways: jasplakinolide and blebbistatin together inhibit the actin cytoskeleton, whereas EIPA inhibits the Na^+^/H^+^ antiporter, and is the nearest to a specific inhibitor for macropinocytosis currently known. CXCL12 only moderately activates Akt and when a more powerful agonist is used — M-CSF acting through a RTK — then the stimulation of Akt is immune to inhibitors of macropinocytosis. However, low doses of M-CSF, giving only a modest activation of Akt, were again sensitive to macropinosome inhibitors. Thus, it appears that doses of agonist giving modest stimulation of Akt are sensitive to macropinocytosis inhibitors, whereas doses giving high Akt activity are not. In other words, forming macropinosomes can somehow amplify signals giving modest activation of Akt, but this amplification is not needed under strong stimulation.

In a recent issue of *Biochemical Journal*, Erami et al. [5] studied Akt activation by the LPA ligand in breast cancer cells. Like CXCL12, LPA acts through a GPCR with coupling achieved by direct interaction of Gβγ with PI3Kβ, leading to PIP3 production and Akt activation. But in a second, more puzzling activation route, expression of activated Rac also leads to increased Akt activity in these cells.

Rac is more normally considered as an activator of actin polymerization rather than PI3K, but in this case it was thought that Rac directly activates PI3Kβ by binding to its Ras-binding domain. However, it was found that mutation of the Ras-binding domain of p110β did not affect Akt activation by Rac, implying that a different route of activation must exist.

Macropinocytosis can be triggered by acute, local activation of Rac [25]. Erami et al. [5] found that expressing activated Rac also stimulates macropinocytosis in their cells, as well as activating Akt and, in an echo of the Pacitto et al. [4] experiments, this Akt activation is sensitive to macropinocytosis inhibitors. Earlier work also suggests that signalling to Akt and Ras is sensitive to cytoskeletal inhibitors [26,27], although without an explicit link to macropinocytosis.

Drawing these studies together, it appears that macropinocytic cups can greatly stimulate weak Akt activation, but are not needed in cases of strong activation [4,5]. Macropinocytic cups, in turn, are shaped around patches of intense PIP3, Ras and Rac signalling fed by a positive feedback, making them sensitive to small stimulations, which they will amplify. Activated Rac can feed in by stimulating actin polymerization and maropinocytosis, and so indirectly activate Akt, while the whole system depends on the physical organization of the macropinosome, making it sensitive to macropinocytic inhibitors (Figure 2).

PIP3 and probably Ras have two faces. They have their familiar roles in signal transduction, but also a less understood role in not just stimulating the actin cytoskeleton, but spatially organizing it into macropinosomes and other structures. A great deal needs to be done at a molecular level to understand how signalling through PI3K is entangled with macropinocytosis, but the origins of this entanglement may date back to our single-celled ancestors in which PIP3 and active Ras were used to organize the actin cytoskeleton into macropinocytic and phagocytic feeding structures, rather than for signal transduction. Since controlling cell feeding and growth is an essential step in establishing multi-cellular organization, these processes may later have fallen under growth-factor control, resulting in the intimate entanglement that we see today.

In summary, where signalling stimulates macropinocytosis, then amplification of sub-maximal levels of PIP3 and possibly active Ras may also be expected, and will, in turn, be sensitive to cytoskeletal and macropinocytic inhibitors.

## Abbreviations

GPCR: G-protein coupled receptor
NF: neurofibromin
PI3K: phosphoinositide 3-kinase
RTKs: tyrosine receptor kinases

## References

[1] Brunk U., Schellens J. and Westermark B. (1976) Influence of epidermal growth factor (EGF) on ruffling activity, pinocytosis and proliferation of cultivated human glia cells. Exp. Cell Res. 103, 295–302 10.1016/0014-4827(76)90266-4 1001364

[2] Bar-Sagi D. and Feramisco J.R. (1986) Induction of membrane ruffling and fluid-phase pinocytosis in quiescent fibroblasts by ras proteins. Science 233, 1061–1068 10.1126/science.3090687 3090687

[3] Wennström S., Hawkins P., Cooke F., Hara K., Yonezawa K., Kasuga M. et al. (1994) Activation of phosphoinositide 3-kinase is required for PDGF-stimulated membrane ruffling. Curr. Biol. 4, 385–393 10.1016/S0960-9822(00)00087-7 7922352

[4] Pacitto R., Gaeta I., Swanson J.A. and Yoshida S. (2017) CXCL12-induced macropinocytosis modulates two distinct pathways to activate mTORC1 in macrophages. J. Leukoc. Biol. 101, 683–692 10.1189/jlb.2A0316-141RR 28250113 PMC5295849

[5] Erami Z., Khalil B.D., Salloum G., Yao Y., LoPiccolo J., Shymanets A. et al. (2017) Rac1-stimulated macropinocytosis enhances Gβγ activation of PI3Kβ. Biochem. J. 474, 3903–3914 10.1042/BCJ20170279 29046393 PMC5858185

[6] Lewis W.H. (1931) Pinocytosis. Bull. Johns Hopkins Hosp. 49, 17–27

[7] Swanson J.A. (2008) Shaping cups into phagosomes and macropinosomes. Nat. Rev. Mol. Cell Biol. 9, 639–649 10.1038/nrm2447 18612320 PMC2851551

[8] Egami Y., Taguchi T., Maekawa M., Arai H. and Araki N. (2014) Small GTPases and phosphoinositides in the regulatory mechanisms of macropinosome formation and maturation. Front. Physiol. 5, 374 10.3389/fphys.2014.00374 25324782 PMC4179697

[9] Yoshida S., Pacitto R., Inoki K. and Swanson J. (2017) Macropinocytosis, mTORC1 and cellular growth control. Cell. Mol. Life Sci. 10.1007/s00018-017-2710-y PMC5843684 29119228

[10] Commisso C., Davidson S.M., Soydaner-Azeloglu R.G., Parker S.J., Kamphorst J.J., Hackett S. et al. (2013) Macropinocytosis of protein is an amino acid supply route in Ras-transformed cells. Nature 497, 633–637 10.1038/nature12138 23665962 PMC3810415

[11] Bloomfield G. and Kay R.R. (2016) Uses and abuses of macropinocytosis. J. Cell Sci. 129, 2697–2705 10.1242/jcs.176149 27352861

[12] Thilo L. and Vogel G. (1980) Kinetics of membrane internalization and recycling during pinocytosis in *Dictyostelium discoideum*. Proc. Natl Acad. Sci. U.S.A. 77, 1015–1019 10.1073/pnas.77.2.1015 6928656 PMC348414

[13] Kayman S.C. and Clarke M. (1983) Relationship between axenic growth of *Dictyostelium discoideum* strains and their track morphology on substrates coated with gold particles. J. Cell Biol. 97, 1001–1010 10.1083/jcb.97.4.1001 6619183 PMC2112609

[14] Hacker U., Albrecht R. and Maniak M. (1997) Fluid-phase uptake by macropinocytosis in *Dictyostelium*. J. Cell Sci. 110(Pt 2), 105–112 PMID:9044041 10.1242/jcs.110.2.105

[15] Bloomfield G., Traynor D., Sander S.P., Veltman D.M., Pachebat J.A. and Kay R.R. (2015) Neurofibromin controls macropinocytosis and phagocytosis in *Dictyostelium*. eLife 4, 10.7554/eLife.04940 PMC4374526 25815683

[16] Araki N., Johnson M.T. and Swanson J.A. (1996) A role for phosphoinositide 3-kinase in the completion of macropinocytosis and phagocytosis by macrophages. J. Cell Biol. 135, 1249–1260 10.1083/jcb.135.5.1249 8947549 PMC2121091

[17] Buczynski G., Grove B., Nomura A., Kleve M., Bush J., Firtel R.A. et al. (1997) Inactivation of two *Dictyostelium discoideum* genes, *DdPIK1* and *DdPIK2*, encoding proteins related to mammalian phosphatidylinositide 3-kinases, results in defects in endocytosis, lysosome to postlysosome transport, and actin cytoskeleton organization. J. Cell Biol. 136, 1271–1286 10.1083/jcb.136.6.1271 9087443 PMC2132510

[18] Hoeller O., Bolourani P., Clark J., Stephens L.R., Hawkins P.T., Weiner O.D. et al. (2013) Two distinct functions for PI3-kinases in macropinocytosis. J. Cell Sci. 126, 4296–4307 10.1242/jcs.134015 23843627 PMC3772393

[19] Yoshida S., Hoppe A.D., Araki N. and Swanson J.A. (2009) Sequential signaling in plasma-membrane domains during macropinosome formation in macrophages. J. Cell Sci. 122, 3250–3261 10.1242/jcs.053207 19690049 PMC2736863

[20] Welliver T.P. and Swanson J.A. (2012) A growth factor signaling cascade confined to circular ruffles in macrophages. Biol. Open 1, 754–760 10.1242/bio.20121784 23213469 PMC3507227

[21] Veltman D.M., Williams T.D., Bloomfield G., Chen B.C., Betzig E., Insall R.H. et al. (2016) A plasma membrane template for macropinocytic cups. eLife 5 10.7554/eLife.20085 PMC5154761 27960076

[22] Dormann D., Weijer G., Dowler S. and Weijer C.J. (2004) *In vivo* analysis of 3-phosphoinositide dynamics during *Dictyostelium* phagocytosis and chemotaxis. J. Cell Sci. 117, 6497–6509 10.1242/jcs.01579 15572406

[23] Welliver T.P., Chang S.L., Linderman J.J. and Swanson J.A. (2011) Ruffles limit diffusion in the plasma membrane during macropinosome formation. J. Cell Sci. 124, 4106–4114 10.1242/jcs.091538 22194306 PMC3244989

[24] Postma M., Roelofs J., Goedhart J., Loovers H.M., Visser A.J. and Van Haastert P.J. (2004) Sensitization of *Dictyostelium* chemotaxis by phosphoinositide-3-kinase-mediated self-organizing signalling patches. J. Cell Sci. 117, 2925–2935 10.1242/jcs.01143 15161938

[25] Fujii M., Kawai K., Egami Y. and Araki N. (2013) Dissecting the roles of Rac1 activation and deactivation in macropinocytosis using microscopic photo-manipulation. Sci. Rep. 3, 2385 10.1038/srep02385 23924974 PMC3737501

[26] Peyrollier K., Hajduch E., Gray A., Litherland G.J., Prescott A.R., Leslie N.R. et al. (2000) A role for the actin cytoskeleton in the hormonal and growth-factor-mediated activation of protein kinase B. Biochem. J. 352, 617–622 10.1042/bj3520617 11104665 PMC1221496

[27] Sperka T., Geißler K.J., Merkel U., Scholl I., Rubio I., Herrlich P. et al. (2011) Activation of Ras requires the ERM-dependent link of actin to the plasma membrane. PLoS ONE 6, e27511 10.1371/journal.pone.0027511 22132106 PMC3221661

[28] Clark J., Kay R.R., Kielkowska A., Niewczas I., Fets L., Oxley D. et al. (2014) *Dictyostelium* uses ether-linked inositol phospholipids for intracellular signalling. EMBO J. 33, 2188–2200 10.15252/embj.201488677 25180230 PMC4282506

